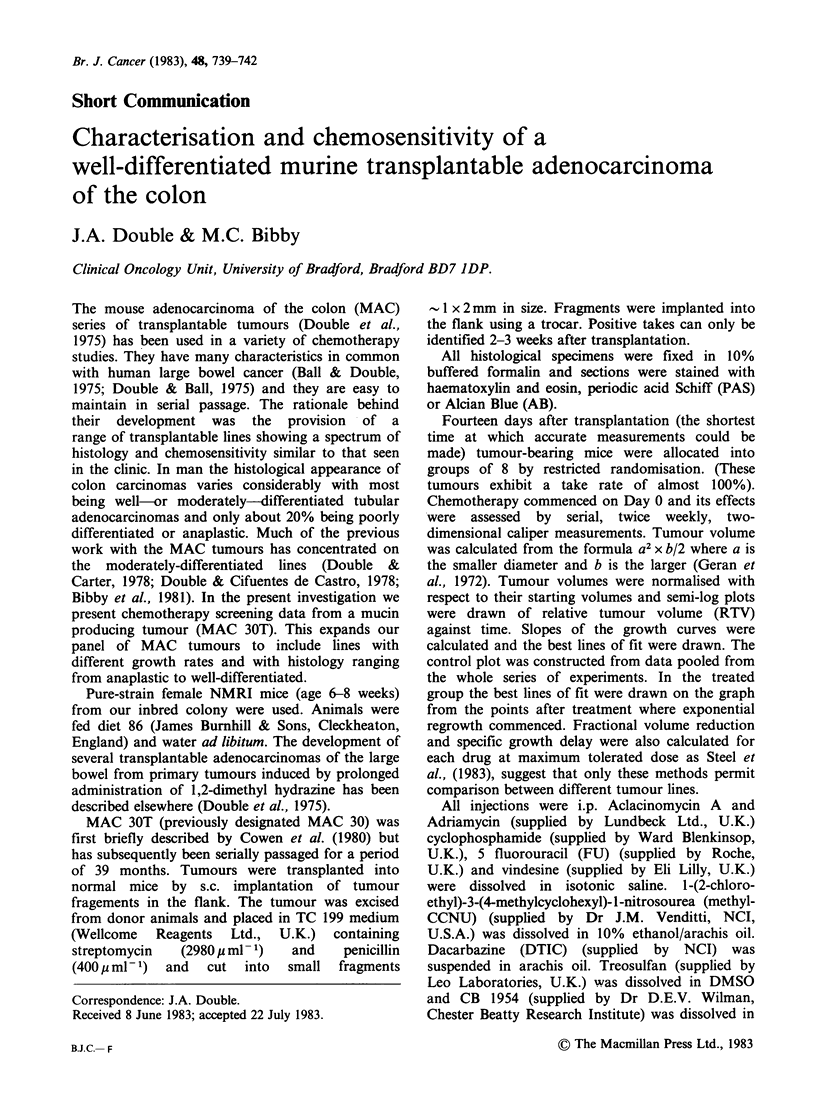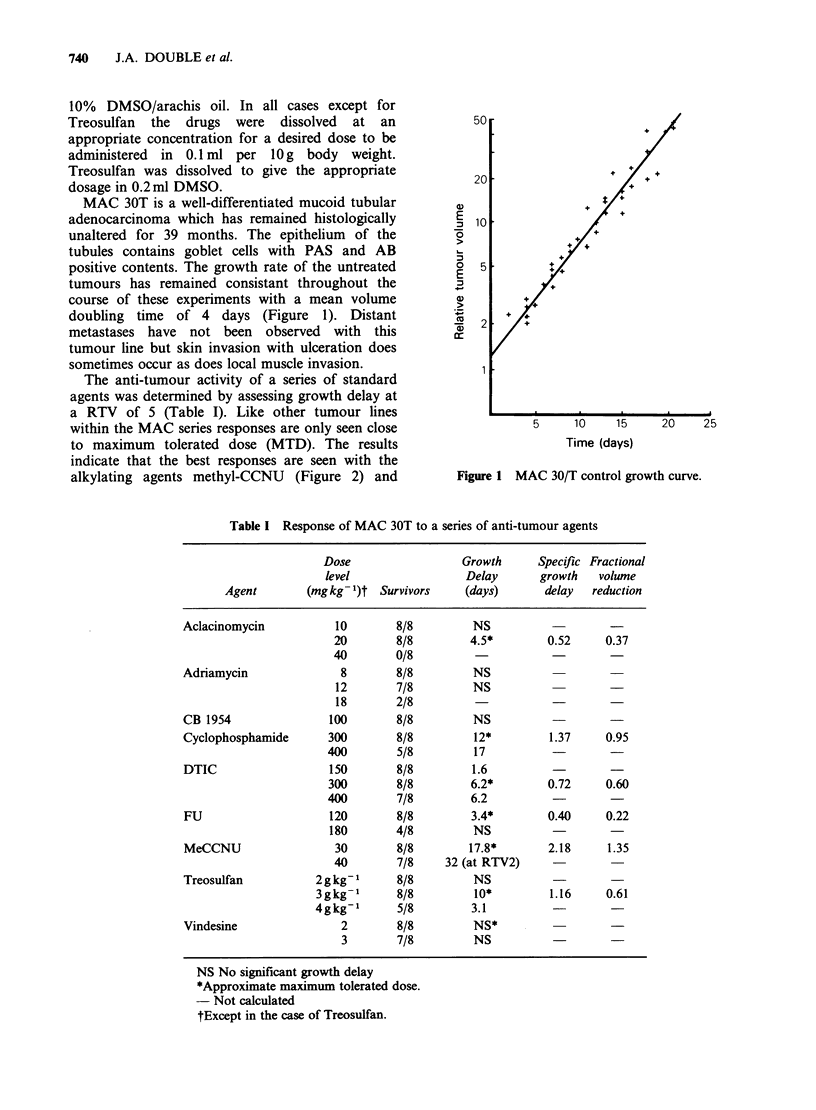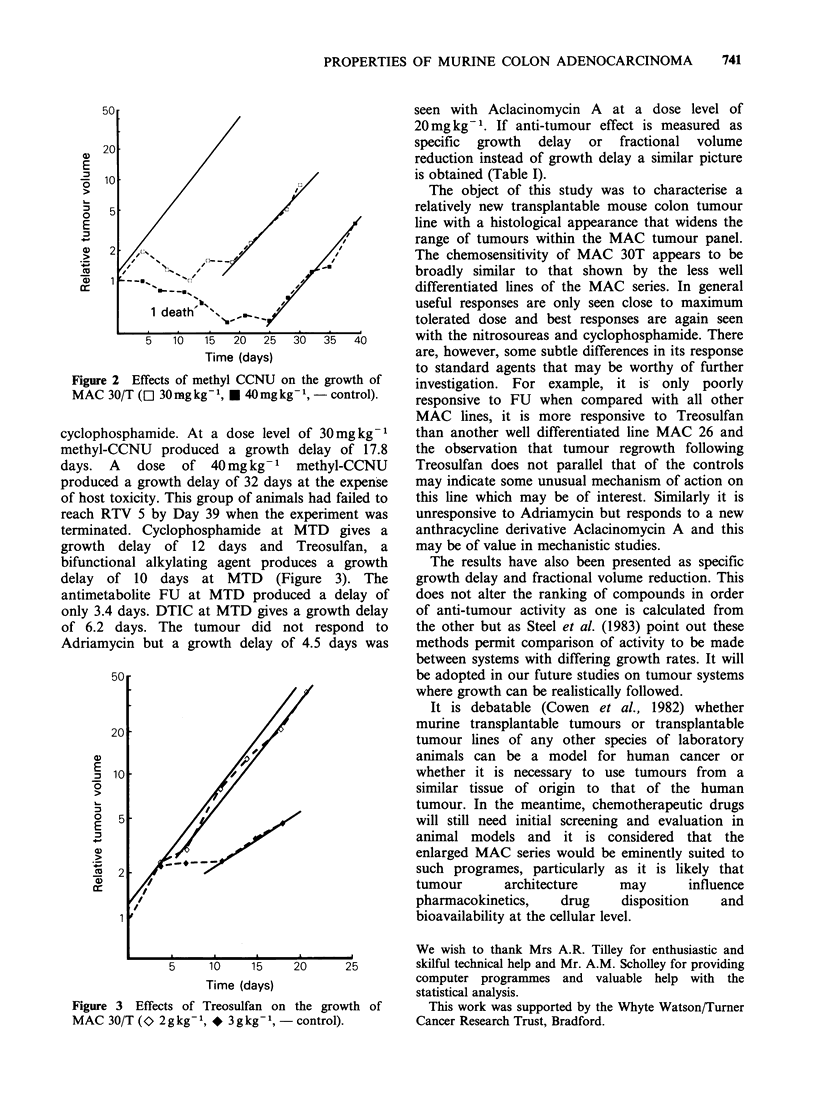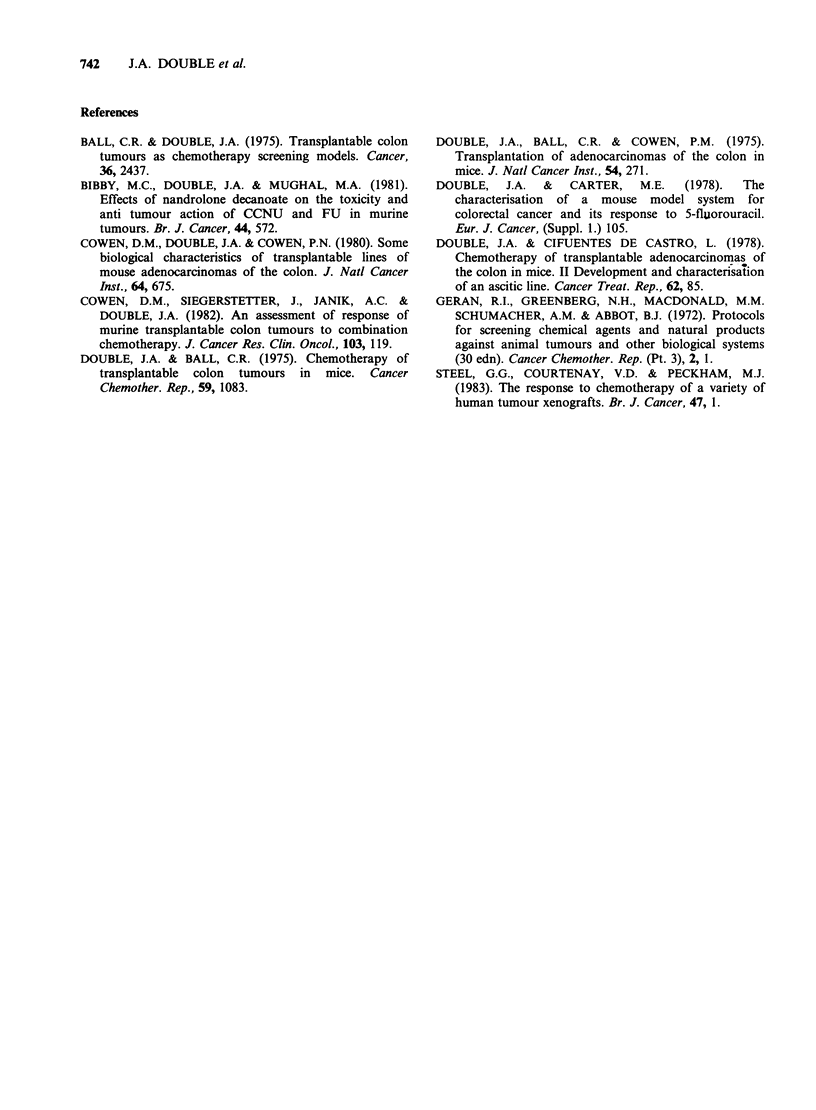# Characterisation and chemosensitivity of a well-differentiated murine transplantable adenocarcinoma of the colon.

**DOI:** 10.1038/bjc.1983.259

**Published:** 1983-11

**Authors:** J. A. Double, M. C. Bibby


*Br. J. Cancer* (1983), **48**, 739–742

**Short Communication**

### Characterisation and chemosensitivity of a well-differentiated murine transplantable adenocarcinoma of the colon

J.A. Double & M.C. Bibby

*Clinical Oncology Unit, University of Bradford, Bradford BD7 1DP.*

The mouse adenocarcinoma of the colon (MAC) series of transplantable tumours (Double *et al.*, 1975) has been used in a variety of chemotherapy studies. They have many characteristics in common with human large bowel cancer (Ball & Double, 1975; Double & Ball, 1975) and they are easy to maintain in serial passage. The rationale behind their development was the provision of a range of transplantable lines showing a spectrum of histology and chemosensitivity similar to that seen in the clinic. In man the histological appearance of colon carcinomas varies considerably with most being well—or moderately—differentiated tubular adenocarcinomas and only about 20% being poorly differentiated or anaplastic. Much of the previous work with the MAC tumours has concentrated on the moderately-differentiated lines (Double & Carter, 1978; Double & Cifuentes de Castro, 1978; Bibby *et al.*, 1981). In the present investigation we present chemotherapy screening data from a mucin producing tumour (MAC 30T). This expands our panel of MAC tumours to include lines with different growth rates and with histology ranging from anaplastic to well-differentiated.

Pure-strain female NMRI mice (age 6–8 weeks) from our inbred colony were used. Animals were fed diet 86 (James Burnhill & Sons, Cleckheaton, England) and water *ad libitum*. The development of several transplantable adenocarcinomas of the large bowel from primary tumours induced by prolonged administration of 1,2-dimethyl hydrazine has been described elsewhere (Double *et al.*, 1975).

MAC 30T (previously designated MAC 30) was first briefly described by Cowen *et al.* (1980) but has subsequently been serially passaged for a period of 39 months. Tumours were transplanted into normal mice by s.c. implantation of tumour fragements in the flank. The tumour was excised from donor animals and placed in TC 199 medium (Wellcome Reagents Ltd., U.K.) containing streptomycin ($2980\,\mu\mathrm{ml}^{-1}$) and penicillin ($400\,\mu\mathrm{ml}^{-1}$) and cut into small fragments $\sim 1\times 2$ mm in size. Fragments were implanted into the flank using a trocar. Positive takes can only be identified 2–3 weeks after transplantation.

All histological specimens were fixed in 10% buffered formalin and sections were stained with haematoxylin and eosin, periodic acid Schiff (PAS) or Alcian Blue (AB).

Fourteen days after transplantation (the shortest time at which accurate measurements could be made) tumour-bearing mice were allocated into groups of 8 by restricted randomisation. (These tumours exhibit a take rate of almost 100%). Chemotherapy commenced on Day 0 and its effects were assessed by serial, twice weekly, two-dimensional caliper measurements. Tumour volume was calculated from the formula $a^2 \times b/2$ where $a$ is the smaller diameter and $b$ is the larger (Geran *et al.*, 1972). Tumour volumes were normalised with respect to their starting volumes and semi-log plots were drawn of relative tumour volume (RTV) against time. Slopes of the growth curves were calculated and the best lines of fit were drawn. The control plot was constructed from data pooled from the whole series of experiments. In the treated group the best lines of fit were drawn on the graph from the points after treatment where exponential regrowth commenced. Fractional volume reduction and specific growth delay were also calculated for each drug at maximum tolerated dose as Steel *et al.*, (1983), suggest that only these methods permit comparison between different tumour lines.

All injections were i.p. Aclacinomycin A and Adriamycin (supplied by Lundbeck Ltd., U.K.) cyclophosphamide (supplied by Ward Blenkinsop, U.K.), 5 fluorouracil (FU) (supplied by Roche, U.K.) and vindesine (supplied by Eli Lilly, U.K.) were dissolved in isotonic saline. 1-(2-chloro-ethyl)-3-(4-methylcyclohexyl)-1-nitrosourea (methyl-CCNU) (supplied by Dr J.M. Venditti, NCI, U.S.A.) was dissolved in 10% ethanol/arachis oil. Dacarbazine (DTIC) (supplied by NCI) was suspended in arachis oil. Treosulfan (supplied by Leo Laboratories, U.K.) was dissolved in DMSO and CB 1954 (supplied by Dr D.E.V. Wilman, Chester Beatty Research Institute) was dissolved in

Correspondence: J.A. Double.
Received 8 June 1983; accepted 22 July 1983.

© The Macmillan Press Ltd., 1983

10% DMSO/arachis oil. In all cases except for Treosulfan the drugs were dissolved at an appropriate concentration for a desired dose to be administered in 0.1 ml per 10 g body weight. Treosulfan was dissolved to give the appropriate dosage in 0.2 ml DMSO.

MAC 30T is a well-differentiated mucoid tubular adenocarcinoma which has remained histologically unaltered for 39 months. The epithelium of the tubules contains goblet cells with PAS and AB positive contents. The growth rate of the untreated tumours has remained consistant throughout the course of these experiments with a mean volume doubling time of 4 days (Figure 1). Distant metastases have not been observed with this tumour line but skin invasion with ulceration does sometimes occur as does local muscle invasion.

The anti-tumour activity of a series of standard agents was determined by assessing growth delay at a RTV of 5 (Table I). Like other tumour lines within the MAC series responses are only seen close to maximum tolerated dose (MTD). The results indicate that the best responses are seen with the alkylating agents methyl-CCNU (Figure 2) and

**Figure 1** MAC 30/T control growth curve.

**Table I** Response of MAC 30T to a series of anti-tumour agents

| *Agent* | *Dose level* $(mg\,kg^{-1})$† | *Survivors* | *Growth Delay (days)* | *Specific growth delay* | *Fractional volume reduction* |
|---|---|---|---|---|---|
| Aclacinomycin | 10 | 8/8 | NS | — | — |
| | 20 | 8/8 | 4.5* | 0.52 | 0.37 |
| | 40 | 0/8 | — | — | — |
| Adriamycin | 8 | 8/8 | NS | — | — |
| | 12 | 7/8 | NS | — | — |
| | 18 | 2/8 | — | — | — |
| CB 1954 | 100 | 8/8 | NS | — | — |
| Cyclophosphamide | 300 | 8/8 | 12* | 1.37 | 0.95 |
| | 400 | 5/8 | 17 | — | — |
| DTIC | 150 | 8/8 | 1.6 | — | — |
| | 300 | 8/8 | 6.2* | 0.72 | 0.60 |
| | 400 | 7/8 | 6.2 | — | — |
| FU | 120 | 8/8 | 3.4* | 0.40 | 0.22 |
| | 180 | 4/8 | NS | — | — |
| MeCCNU | 30 | 8/8 | 17.8* | 2.18 | 1.35 |
| | 40 | 7/8 | 32 (at RTV2) | — | — |
| Treosulfan | $2\,g\,kg^{-1}$ | 8/8 | NS | — | — |
| | $3\,g\,kg^{-1}$ | 8/8 | 10* | 1.16 | 0.61 |
| | $4\,g\,kg^{-1}$ | 5/8 | 3.1 | — | — |
| Vindesine | 2 | 8/8 | NS* | — | — |
| | 3 | 7/8 | NS | — | — |

NS No significant growth delay
*Approximate maximum tolerated dose.
— Not calculated
†Except in the case of Treosulfan.

**Figure 2** Effects of methyl CCNU on the growth of MAC 30/T (□ 30 mg kg$^{-1}$, ■ 40 mg kg$^{-1}$, — control).

cyclophosphamide. At a dose level of 30 mg kg$^{-1}$ methyl-CCNU produced a growth delay of 17.8 days. A dose of 40 mg kg$^{-1}$ methyl-CCNU produced a growth delay of 32 days at the expense of host toxicity. This group of animals had failed to reach RTV 5 by Day 39 when the experiment was terminated. Cyclophosphamide at MTD gives a growth delay of 12 days and Treosulfan, a bifunctional alkylating agent produces a growth delay of 10 days at MTD (Figure 3). The antimetabolite FU at MTD produced a delay of only 3.4 days. DTIC at MTD gives a growth delay of 6.2 days. The tumour did not respond to Adriamycin but a growth delay of 4.5 days was

**Figure 3** Effects of Treosulfan on the growth of MAC 30/T (◇ 2 g kg$^{-1}$, ◆ 3 g kg$^{-1}$, — control).

seen with Aclacinomycin A at a dose level of 20 mg kg$^{-1}$. If anti-tumour effect is measured as specific growth delay or fractional volume reduction instead of growth delay a similar picture is obtained (Table I).

The object of this study was to characterise a relatively new transplantable mouse colon tumour line with a histological appearance that widens the range of tumours within the MAC tumour panel. The chemosensitivity of MAC 30T appears to be broadly similar to that shown by the less well differentiated lines of the MAC series. In general useful responses are only seen close to maximum tolerated dose and best responses are again seen with the nitrosoureas and cyclophosphamide. There are, however, some subtle differences in its response to standard agents that may be worthy of further investigation. For example, it is only poorly responsive to FU when compared with all other MAC lines, it is more responsive to Treosulfan than another well differentiated line MAC 26 and the observation that tumour regrowth following Treosulfan does not parallel that of the controls may indicate some unusual mechanism of action on this line which may be of interest. Similarly it is unresponsive to Adriamycin but responds to a new anthracycline derivative Aclacinomycin A and this may be of value in mechanistic studies.

The results have also been presented as specific growth delay and fractional volume reduction. This does not alter the ranking of compounds in order of anti-tumour activity as one is calculated from the other but as Steel *et al.* (1983) point out these methods permit comparison of activity to be made between systems with differing growth rates. It will be adopted in our future studies on tumour systems where growth can be realistically followed.

It is debatable (Cowen *et al.*, 1982) whether murine transplantable tumours or transplantable tumour lines of any other species of laboratory animals can be a model for human cancer or whether it is necessary to use tumours from a similar tissue of origin to that of the human tumour. In the meantime, chemotherapeutic drugs will still need initial screening and evaluation in animal models and it is considered that the enlarged MAC series would be eminently suited to such programes, particularly as it is likely that tumour architecture may influence pharmacokinetics, drug disposition and bioavailability at the cellular level.

We wish to thank Mrs A.R. Tilley for enthusiastic and skilful technical help and Mr. A.M. Scholley for providing computer programmes and valuable help with the statistical analysis.

This work was supported by the Whyte Watson/Turner Cancer Research Trust, Bradford.

### References

BALL, C.R. & DOUBLE, J.A. (1975). Transplantable colon tumours as chemotherapy screening models. *Cancer*, **36**, 2437.

BIBBY, M.C., DOUBLE, J.A. & MUGHAL, M.A. (1981). Effects of nandrolone decanoate on the toxicity and anti tumour action of CCNU and FU in murine tumours. *Br. J. Cancer*, **44**, 572.

COWEN, D.M., DOUBLE, J.A. & COWEN, P.N. (1980). Some biological characteristics of transplantable lines of mouse adenocarcinomas of the colon. *J. Natl Cancer Inst.*, **64**, 675.

COWEN, D.M., SIEGERSTETTER, J., JANIK, A.C. & DOUBLE, J.A. (1982). An assessment of response of murine transplantable colon tumours to combination chemotherapy. *J. Cancer Res. Clin. Oncol.*, **103**, 119.

DOUBLE, J.A. & BALL, C.R. (1975). Chemotherapy of transplantable colon tumours in mice. *Cancer Chemother. Rep.*, **59**, 1083.

DOUBLE, J.A., BALL, C.R. & COWEN, P.M. (1975). Transplantation of adenocarcinomas of the colon in mice. *J. Natl Cancer Inst.*, **54**, 271.

DOUBLE, J.A. & CARTER, M.E. (1978). The characterisation of a mouse model system for colorectal cancer and its response to 5-fluorouracil. *Eur. J. Cancer*, (Suppl. 1.) 105.

DOUBLE, J.A. & CIFUENTES DE CASTRO, L. (1978). Chemotherapy of transplantable adenocarcinomas of the colon in mice. II Development and characterisation of an ascitic line. *Cancer Treat. Rep.*, **62**, 85.

GERAN, R.I., GREENBERG, N.H., MACDONALD, M.M. SCHUMACHER, A.M. & ABBOT, B.J. (1972). Protocols for screening chemical agents and natural products against animal tumours and other biological systems (30 edn). *Cancer Chemother. Rep.* (Pt. 3), **2**, 1.

STEEL, G.G., COURTENAY, V.D. & PECKHAM, M.J. (1983). The response to chemotherapy of a variety of human tumour xenografts. *Br. J. Cancer*, **47**, 1.